# Associations of CYP1 polymorphisms with risk of prostate cancer: an updated meta-analysis

**DOI:** 10.1042/BSR20181876

**Published:** 2019-03-01

**Authors:** Wei Zhu, Hailang Liu, Xinguang Wang, Jinjin Lu, Huiping Zhang, Shaogang Wang, Weimin Yang

**Affiliations:** 1 Department of Urology, Tongji Hospital, Tongji Medical College, Huazhong University of Science and Technology, Wuhan 430030, Hubei, China; 2 Institute of Family Planning Research, Tongji Medical College, Huazhong University of Science and Technology, Wuhan 430030, Hubei, China

**Keywords:** CYP1A1, CYP1B1, meta-analysis, polymorphisms, prostate cancer

## Abstract

**Background.** The results of previous studies on the association between polymorphisms of CYP1A1 and CYP1B1 and prostate cancer (PCa) susceptibility are inconsistent. The aim of the present study was to conduct a meta-analysis in order to better estimate this association. **Methods.** A systematic search was carried out on PubMed, Embase, Cochrane Library, and China National Knowledge Infrastructure (CNKI) databases for relevant articles published up to 15 August 2018. Pooled odds ratios (ORs) and 95% confidence intervals were obtained using fixed-effect or random-effect models. **Results.** A significant association was found between the CYP1A1 rs1048943 polymorphism and PCa in the overall population (B [the minor allele] vs. A [the major allele]: OR = 1.20, 95% confidence interval (CI) = 1.04–1.39, *P*=0.014; AB vs. AA: OR = 1.24, 95% CI = 1.02–1.51, *P*=0.029; BB + AB vs. AA: OR = 1.25, 95% CI = 1.04–1.50, *P*=0.018) and Asian population (B vs. A: OR = 1.32, 95% CI = 1.11–1.56, *P*=0.001; BB vs. AA: OR = 1.81, 95% CI = 1.20–2.72, *P*=0.005; AB vs. AA: OR = 1.30, 95% CI = 1.03–1.64, *P*=0.029; BB + AB vs. AA: OR = 1.38, 95% CI = 1.11–1.73, *P*=0.004; BB vs. AA + AB: OR = 1.58, 95% CI = 1.08–2.01, *P*=0.019), but not in the Caucasian population. Moreover, we found that the rs4646903 polymorphism was associated with a significant increase in the risk of PCa in the Asian population (AB vs. AA: OR = 1.43, 95% CI = 1.13–1.80, *P*=0.003) and Caucasian population (BB vs. AA: OR = 2.12, 95% CI = 1.29–3.49, *P*=0.003). **Conclusion.** This meta-analysis revealed a clear association between rs1048943 and rs4646903 polymorphisms of the *CYP1A1* gene but not between CYP1B1 rs10012, rs162549, rs1800440, and rs2551188 polymorphisms and the risk of PCa.

## Introduction

Prostate cancer (PCa) is the most common malignant tumor of the urinary system in men in the western world, the second most frequent cancer and the fifth leading cause of tumor-related deaths worldwide [1,2]. Globally, it is estimated that there will be approximately 1.3 million newly diagnosed cases of PCa and 359000 associated deaths in 2018 [1], making the disease a major health problem in men. The occurrence and development of PCa is influenced by both environmental and genetic factors. Risk factors associated with PCa include age, lifestyle, familial heredity, and hormonal status, but the exact etiology is unclear [3]. It has been shown that exposure to aromatic hydrocarbons is associated with a high risk of PCa [4], and racial differences and familial aggregation are amongst the genetic factors that influence the incidence and development of the disease. Therefore, an increasing number of studies are attempting to explain the occurrence, development, and prognosis of PCa through analysis of variations in the genes involved in hormone metabolism.

A single nucleotide polymorphism (SNP) is a single nucleotide variation at the genomic level, which can appear in coding or non-coding sequences. In recent years, third-generation genetic markers have attracted a lot of attention for their potential role in predicting cancer susceptibility. A number of genes including those encoding the androgen receptor (AR), prostate-specific antigen (PSA), 5a-reductase type II (SRD5A2), and cytochrome P450 (CYP) have been confirmed as PCa susceptibility genes [4]. The CYPs are a large superfamily of conserved proteins found in animals, plants, and microorganisms. Cytochrome P450 1A1 (CYP1A1) and 1B1 (CYP1B1) are pivotal members of the CYP1 family. Classified as a phase I enzyme, CYP1A1 is involved in aryl hydrocarbon hydroxylase activity, while CYP1B1 participates in hydroxylation of estrogens. He and Feng [5] reported that CYP1A1 is involved in the metabolism of estrogen and also activates procarcinogens. Furthermore, it has been shown that the CYP1A1 polymorphism is associated with PCa development [6]. Tokizane et al. [7] found that hypomethylation of the promoter/enhancer region leads to overexpression of CYP1B1 in PCa cells, and metabolites from CYP1B1 catalysis have been demonstrated to induce PCa in an animal model [8]. As an androgen-dependent organ, the prostate contains several enzymes, including CYP1A1 and CYP1B1, which participate in the metabolism of steroid hormones. It is evident from these reports that CYP1A1 and CYP1B1 play crucial roles in the development of PCa and are potential diagnostic and predictive markers, as well as therapeutic targets.

Previous meta-analyses investigating the relationship between CYP1A1 and CYP1B1 polymorphisms and the risk of PCa reported inconsistent results. Cui et al. [9] did not find the CYP1B1 rs1056836 polymorphism to be associated with PCa, while another study found the CYP1A1 rs1048943, but not rs4646903, polymorphism to be associated with a high risk of PCa [10]. Another study on an Indian cohort reported the CYP1A1 rs1048943 polymorphism to be associated with a lower risk of PCa, while rs4646903 was related to a high PCa risk [11]. Although these findings strongly suggest that CYP1A1 and CYP1B1 polymorphisms may be closely related to the susceptibility to PCa, they do not provide an across-the-board estimate of the association. In the past few years, attempts to define the exact relationship have been stymied because of differences in study designs, genotyping methods, and the ethnicity and genetics of the study populations. Therefore, the present meta-analysis, which includes previous and some recently published studies, was undertaken to gain a more precise estimation of the association of polymorphisms with PCa, and to evaluate the possible risk factors.

## Materials and methods

### Literature search strategy

The PubMed, Embase, Cochrane Library, and China National Knowledge Infrastructure (CNKI) databases were systematically searched independently by two authors (W.Z. and H.L.) on 15 August 2018 to retrieve eligible articles. The search terms included: ‘cytochrome P450 1A1’, ‘cytochrome P450 1B1’, ‘CYP1A1’, ‘CYP1B1’, ‘prostate cancer’, ‘PCa’, ‘PC’, ‘gene’, ‘polymorphism’, ‘allele’ and ‘variation’. In addition, reference lists of the retrieved papers were manually reviewed to obtain additional studies that met the inclusion criteria.

### Study selection criteria

Studies selected for the meta-analysis had to meet the following inclusion criteria: (i) the study investigated the association between CYP1A1, CYP1B1 polymorphism, and risk of PCa, (ii) the design was a case–control study, (iii) sufficient data were reported to determine the pooled odds ratios (ORs) and 95% confidence intervals (95% CIs), and (iv) the full text was published. Meta-analyses, letters, single-case reports, duplicate studies, animal model studies, and studies without available data were excluded.

### Data extraction

Two authors (W.Z. and X.W.) independently extracted the available data from included papers, and any disagreements were resolved by the third investigator (J.L.). The following information was extracted and recorded: author, year of publication, ethnicity of subjects (Asian, African, Caucasian, or mixed), the name of gene, the names of SNPs, genotyping method (PCR-restriction fragment length polymorphism [PCR-RFLP], Taqman, allele-specific PCR [AS-PCR] or Genechips), source of controls (population or hospital), total numbers of cases and controls, and the Newcastle–Ottawa Scale (NOS). Studies which investigated more than one kind of SNP were counted as individual data for the present meta-analysis. The quality of all included studies was assessed independently by the three researchers using the validated NOS, and disagreements were resolved through discussion with another researcher (H.Z.). Studies with scores of >6 were considered as high-quality studies, and those with scores of ≤6 as low-quality studies.

### Statistical analysis

We utilized Stata 12.0 software (Stata Corp LP, College Station, TX) to conduct the meta-analysis and estimate heterogeneity between the included studies. ORs and 95% confidence intervals (CIs) were used to assess any associations between CYP1A1 and CYP1B1 polymorphisms and the risk of PCa. The pooled ORs were calculated in BB vs. AA (A: the major allele, B: the minor allele), BB + AB vs. AA, BB vs. AB + AA, B vs. A, and AB vs. AA genetic models using either the fixed-effects model or the random-effect model. In case of significant heterogeneity (*Q*-test: *I*^2^ ≥ 50% or *P*≤0.05) between studies, a random-effect model was used for analysis; otherwise, a fixed-effect model would be chosen [12]. Subgroup analyses were performed based on ethnicity (Caucasian, Asian, African, or mixed). Finally, sensitivity analysis was performed to examine whether the independent studies included in the meta-analysis affected the pooled results, and publication bias was evaluated by Egger’s test [13].

## Results

### Characteristics of included studies

**Figure 1:**
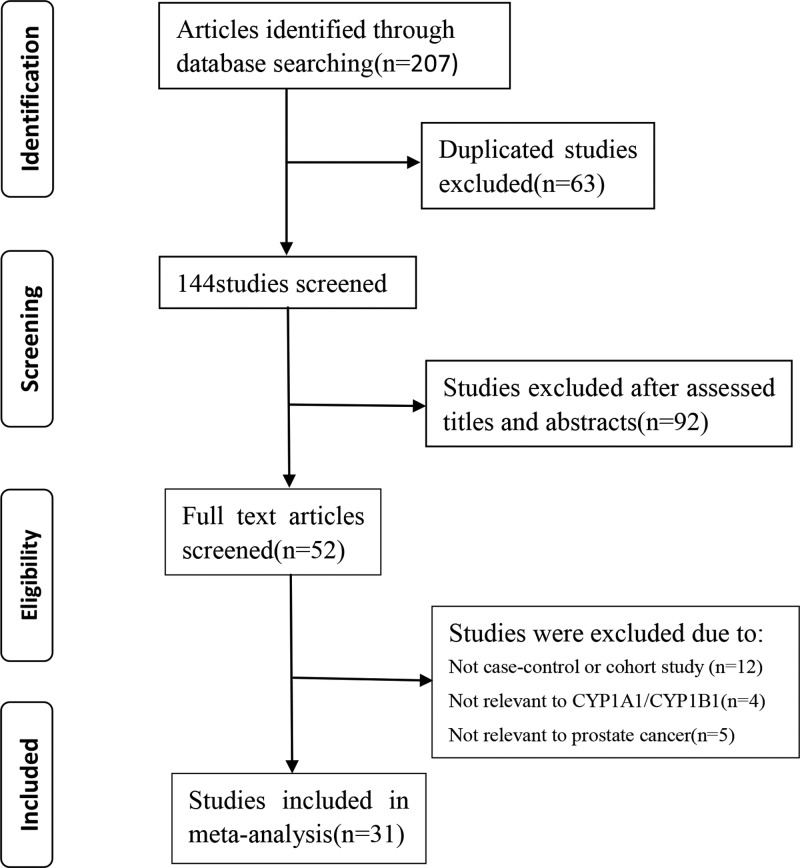
The flow diagram of this meta-analysis

A flow diagram of the study selection is shown in Figure 1. We retrieved 207 studies from the PubMed, Embase, Cochrane Library, and CNKI databases. Initial screening excluded 63 duplicated articles, and 92 papers that did not fit the inclusion criteria were excluded after careful review of the titles and abstracts. Out of the 52 studies selected for full publication review, 21 were excluded (12 studies were not case–control studies and 9 were not related to CYP1A1/CYP1B1 or PCa). Finally, 31 articles [11,14–43] including 12745 cases and 12471 controls that met the inclusion criteria were included in the final analysis. The main characteristics of these studies are summarized in Table 1. Genotype frequency was available in all included studies. A total of 16 Caucasian, 12 Asian, 1 African, and 2 mixed populations were represented in the 31 selected studies. Of note, three SNPs in CYP1A1 and twelve in CYP1B1 were excluded from the final analysis due to lack of sufficient research.

**Table 1:** Characteristics of the studies included in the meta-analysis

Author	Year	Ethnicity	Gene	SNP	Genotyping method	Source of control	Cases/controls	NOS
Tanaka et al.	2002	Asian	CYP1B1	rs1056827, rs2551188, rs10012, rs1056836, rs1056837	PCR-RFLP	Hospital	117/200	8
Murata et al.	2001	Asian	CYP1A1	rs1048943	PCR-RFLP	Hospital	115/200	7
Suzuki et al.	2003	Asian	CYP1A1	rs4646903, rs1048943	PCR-RFLP	Hospital	81/105	8
Mandić et al.	2014	Caucasian	CYP1A1	rs4646903	Taqman	Hospital	120/120	7
Fukatsu et al.	2004	Asian	CYP1B1	rs1056836	PCR-RFLP	Population	147/266	7
Acevedo et al.	2003	Caucasian	CYP1A1	rs4646903	PCR-RFLP	Population	102/102	6
Aktas et al.	2004	Caucasian	CYP1A1	rs4986883	AS-PCR	Hospital	100/107	7
Beer et al.	2002	Caucasian	CYP1A1	rs1048943	PCR-RFLP	Population	117/183	7
Beuten et al.	2008	Caucasian	CYP1B1	rs2567206, rs2551188, rs2617266, rs10012, rs1056836, rs1800440	Taqman	Population	649/738	8
Brureau et al.	2016	Mixed	CYP1B1	rs1056836	PCR-RFLP	Population	660/709	7
Cáceres et al.	2005	Caucasian	CYP1A1	rs4646903	PCR-RFLP	Population	103/132	6
Vijayalakshmi et al.	2005	Asian	CYP1A1	rs4646903, rs1048943	PCR-RFLP	Population	50/50	7
Catsburg et al.	2012	Caucasian	CYP1B1	rs1056836	Taqman	Population	1343/800	9
Cicek et al.	2005	Caucasian	CYP1B1	rs1056827, rs1056836	PCR-RFLP	Population	439/479	8
Cusseno et al.	2007	Caucasian	CYP1B1	rs1056836	Taqman	Population	1101/882	6
Lima et al.	2008	Caucasian	CYP1A1	rs4646903	PCR-RFLP	Hospital	125/100	7
Holt et al.	2012	Caucasian	CYP1A1/CYP1B1	rs162549, rs2855658, rs1056836, rs1456432, rs4886605	PCR-RFLP	Population	1304/1266	8
Gu et al.	2018	Asian	CYP1B1	rs9341266, rs162549, rs10916, rs162562, rs2551188, rs9341250, rs1056827, rs1056836	Taqman	Population	1015/1052	7
Kachakova et al.	2016	Caucasian	CYP1B1	rs1056836, rs1056837, rs1800440, rs1056827,	PCR-RFLP	Hospital	246/261	6
Kato et al.	2018	Caucasian	CYP1B1	rs2551188, rs2567206, rs2567207, rs162556, rs10175368, rs163090, rs162330, rs162331	Taqman	Population	400/405	8
Kumar et al.	2010	Asian	CYP1A1	rs4646903, rs1048943	PCR-RFLP	Population	70/61	6
Mittal et al.	2007	Asian	CYP1A1	rs4646903	PCR-RFLP	Population	130/140	6
Nock et al.	2006	Mixed	CYP1A1	rs1048943	PCR-RFLP	Population	439/479	6
Price et al.	2016	Caucasian	CYP1B1	rs1800440	Taqman	Population	1506/1380	9
Quiñones et al.	2006	Caucasian	CYP1A1	rs4646903	PCR-RFLP	Population	60/117	6
Rodrigues et al.	2010	Caucasian	CYP1A1/CYP1B1	rs1048943, rs1056827	AS-PCR	Population	154/154	6
Sobti et al.	2006	Asian	CYP1B1	rs1056836	PCR-RFLP	Population	100/100	7
Souiden et al.	2012	African	CYP1A1	rs4646903	PCR-RFLP	Population	138/138	6
Tang et al.	2017	Asian	CYP1B1	rs10012, rs1800440	Taqman	Population	1506/1380	8
Yang et al.	2006	Asian	CYP1A1	rs4646903, rs1048943	AS-PCR	Hospital	225/250	6
Guan et al.	2005	Asian	CYP1A1	rs4646903, rs1048943	Genechips	Hospital	83/115	6

### Meta-analysis

**Figure 2:**
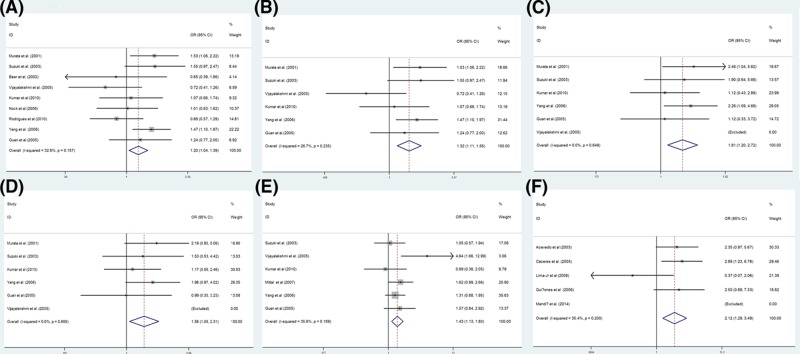
Forest plot for the association between gene polymorphisms and PCa Forest plot for rs1048943 polymorphism in overall population (B vs. A, (**A**)) and Asian population (B vs. A, (**B**); BB vs. AA, (**C**); BB vs. AA+AB, (**D**)), for rs4646903 polymorphism in Asian population (AB vs. AA, (**E**)) and Caucasian population (BB vs. AA, (**F**)). Abbreviation: vs., versus.

The results of the meta-analysis are shown in Table 2. Meanwhile, the forest plots for positive results are shown in Figure 2.

**Table 2:** Meta-analysis of CYP1 gene polymorphisms and PCa

Polymorphisms	Comparisons	Number of studies	Test of association				Heterogeneity	
			OR (95% CI)	Z	*P*-value	Model	*P*-value	*I^2^* (%)
CYP1A1								
rs1048943								
Overall	B vs. A	9	1.20 (1.04, 1.39)	2.46	0.014	F	0.157	32.6
	BB vs. AA	9	1.37 (0.97, 1.93)	1.8	0.072	F	0.122	38.6
	AB vs. AA	9	1.24 (1.02, 1.51)	2.15	0.029	F	0.11	38.8
	BB + AB vs. AA	9	1.25 (1.04, 1.50)	2.37	0.018	F	0.132	35.8
	BB vs. AA + AB	9	1.21 (0.92, 1.61)	1.35	0.178	F	0.135	36.9
Asian	B vs. A	6	1.32 (1.11, 1.56)	3.23	0.001	F	0.235	26.7
	BB vs. AA	5	1.81 (1.20, 2.72)	2.84	0.005	F	0.649	0
	AB vs. AA	6	1.30 (1.03, 1.64)	2.19	0.029	F	0.151	38.3
	BB + AB vs. AA	6	1.38 (1.11, 1.73)	2.87	0.004	F	0.129	41.5
	BB vs. AA + AB	6	1.58 (1.08, 2.01)	2.38	0.019	F	0.699	0
Caucasian	B vs. A	2	0.86 (0.60, 1.23)	0.83	0.405	F	0.984	0
	BB vs. AA	2	0.66 (0.31, 1.37)	1.12	0.262	R	0.114	60
	AB vs. AA	2	1.16 (0.71, 1.89)	0.59	0.557	R	0.038	76.8
	BB + AB vs. AA	2	0.97 (0.63, 1.49)	0.15	0.883	F	0.33	0
	BB vs. AA + AB	2	0.63 (0.32, 1.22)	1.36	0.172	R	0.1	63.1
rs4646903								
Overall	B vs. A	12	1.18 (1.04, 1.33)	2.62	0.009	R	0.001	65.7
	BB vs. AA	11	1.16 (0.88, 1.53)	1.07	0.286	F	0.111	35.9
	AB vs. AA	12	1.41 (1.19, 1.68)	3.99	<0.001	R	0.007	57.5
	BB + AB vs. AA	12	1.36 (1.15, 1.59)	3.69	<0.001	R	0.001	65
	BB vs. AA + AB	12	0.95 (0.74, 1.23)	0.36	0.72	F	0.458	0
Asian	B vs. A	6	1.07 (0.92, 1.26)	0.88	0.377	R	0.038	57.6
	BB vs. AA	6	0.88 (0.63, 1.24)	0.7	0.482	F	0.791	0
	AB vs. AA	6	1.43 (1.13, 1.80)	2.99	0.003	F	0.169	35.6
	BB + AB vs. AA	6	1.30 (1.04, 1.61)	2.31	0.021	R	0.068	51.3
	BB vs. AA + AB	6	0.79 (0.58, 1.07)	1.52	0.128	F	0.901	0
Caucasian	B vs. A	5	1.43 (1.16, 1.75)	3.41	0.001	R	0.007	71.9
	BB vs. AA	4	2.12 (1.29, 3.49)	2.97	0.003	F	0.2	35.4
	AB vs. AA	5	1.54 (1.17, 2.02)	3.08	0.002	R	0.004	74
	BB + AB vs. AA	5	1.57 (1.21, 2.04)	3.38	0.001	R	0.001	77.5
	BB vs. AA + AB	5	1.49 (0.93, 2.37)	1.67	0.095	F	0.366	5.4
CYP1B1								
rs1056836								
Overall	B vs. A	12	1.02 (0.96, 1.07)	0.61	0.54	R	<0.001	73.4
	BB vs. AA	12	1.04 (0.93, 1.17)	0.65	0.514	R	<0.001	70.5
	AB vs. AA	12	1.03 (0.96, 1.12)	0.84	0.401	F	0.077	39.5
	BB + AB vs. AA	12	1.03 (0.96, 1.11)	0.77	0.441	R	0.002	63.1
	BB vs. AA + AB	12	1.01 (0.91, 1.12)	0.1	0.916	R	0.002	62.2
Asian	B vs. A	4	0.99 (0.87, 1.14)	0.09	0.932	R	<0.001	84.6
	BB vs. AA	4	1.20 (0.78, 1.85)	0.85	0.398	R	0.004	77.1
	AB vs. AA	4	0.96 (0.81, 1.12)	0.54	0.587	R	0.026	67.7
	BB + AB vs. AA	4	0.97 (0.83, 1.14)	0.36	0.722	R	0.002	79.4
	BB vs. AA + AB	4	1.17 (0.76, 1.79)	0.71	0.477	R	0.012	72.8
Caucasian	B vs. A	7	1.02 (0.96, 1.09)	0.78	0.433	R	0.002	72
	BB vs. AA	7	1.03 (0.91, 1.16)	0.45	0.649	R	0.001	74.8
	AB vs. AA	7	1.07 (0.97, 1.17)	1.39	0.163	R	0.314	15.2
	BB + AB vs. AA	7	1.05 (0.97, 1.15)	1.19	0.234	R	0.03	57
	BB vs. AA + AB	7	1.00 (0.90, 1.11)	0.06	0.951	R	0.007	66.1
rs10012								
Overall	G vs. C	3	1.05 (0.91, 1.21)	0.66	0.508	F	0.545	0
rs162549								
Overall	T vs. A	2	1.07 (0.96, 1.19)	1.21	0.226	F	0.515	0
rs1056827								
Overall	T vs. G	5	1.14 (1.03, 1.26)	2.47	0.014	R	<0.001	84
rs1056837								
Overall	T vs. C	2	0.90 (0.74, 1.11)	0.96	0.338	R	0.136	54.9
rs1800440								
Overall	G vs. A	4	0.95 (0.85, 1.06)	0.89	0.374	F	0.477	0
rs2551188								
Overall	T vs. C	4	0.96 (0.87, 1.07)	0.72	0.474	F	0.873	0
rs2567206								
Overall	T vs. C	2	1.21 (1.01, 1.45)	2.03	0.042	R	0.077	68

Abbreviations: F, fixed-effect model; R, random-effect model; vs., versus.

#### Analysis of the CYP1A1 rs1048943 polymorphism

Overall, analysis of the association between the rs1048943 polymorphism and PCa from nine independent studies revealed no significant heterogeneity by a fixed-effect model. The results, however, showed a significant association between the rs1048943 polymorphism and PCa after comparing the B allele vs. A allele, AB vs. AA and BB + AB vs. AA (OR = 1.20, 95% CI = 1.04–1.39, *P*=0.014; OR = 1.24, 95% CI = 1.02–1.51, *P*=0.029; OR = 1.25, 95% CI = 1.04–1.50, *P*=0.018; respectively). Meanwhile, no association was evident when examining BB vs. AA and BB vs. AA + AB (OR = 1.37, 95% CI = 0.97–1.93, *P*=0.072; OR = 1.21, 95% CI = 0.92–1.61, *P*=0.178; respectively).

The *Q*-test revealed no significant heterogeneity in the Asian populations when analyzed from six independent studies. Therefore, we conducted an analysis using the fixed-effect model which showed a significant association between the rs1048943 polymorphism and PCa when comparing the B allele vs. A allele, BB vs. AA, AB vs. AA, BB + AB vs. AA, and BB vs. AA + AB (OR = 1.32, 95% CI = 1.11–1.56, *P*=0.001; OR = 1.81, 95% CI = 1.20–2.72, *P*=0.005; OR = 1.30, 95% CI = 1.03–1.64, *P*=0.029; OR = 1.38, 95% CI = 1.11–1.73, *P*=0.004; OR = 1.58, 95% CI = 1.08–2.01, *P*=0.019; respectively).

In the Caucasian population, significant between-study heterogeneity was found when comparing BB vs. AA, AB vs. AA, BB vs. AA + AB by the random-effect model. There was no significant heterogeneity for B allele vs. A allele or BB + AB vs. AA models, and the results showed no association of the rs1048943 polymorphism with PCa susceptibility (B vs. A, OR = 0.86, 95% CI = 0.60–1.23, *P*=0.405; BB + AB vs. AA, OR = 0.97, 95% CI = 0.63–1.49, *P*=0.883).

#### Analysis of the CYP1A1 gene rs4646903 polymorphism

Overall, significant between-study heterogeneity was detected for most of the genetic models, except for the BB vs. AA and BB vs. AA + AB models. Fixed-effect model analysis of these two genetic models indicated no significant association (BB vs. AA, OR = 1.16, 95% CI = 0.88–1.53, *P*=0.286; BB vs. AA + AB, OR = 0.95, 95% CI = 0.74–1.23, *P*=0.72).

In the Asian populations, there was significant between-study heterogeneity in the B allele vs. A allele and BB + AB vs. AA models, and the results showed an association between rs4646903 and PCa when comparing AB vs. AA (OR = 1.43, 95% CI = 1.13–1.80, *P*=0.003), which was not evident when comparing BB vs. AA (OR = 0.88, 95% CI = 0.63–1.24, *P*=0.482) or BB vs. AA + AB (OR = 0.79, 95% CI = 0.58–1.07, *P*=0.128).

In the Caucasian populations, significant between-study heterogeneity was found in the B allele vs. A allele, AB vs. AA, and BB + AB vs. AA models. There was a significant association between rs4646903 and PCa when comparing BB vs. AA (OR = 2.12, 95% CI = 1.29–3.49, *P*=0.003). Comparing BB vs. AA + AB, however, indicated no association (OR = 1.49, 95% CI = 0.93–2.37, *P*=0.095).

#### Analysis of the CYP1B1 rs1056836 polymorphism

Except for AB vs. AA in the overall population, significant between-study heterogeneity was found in all other genetic models. However, the association between the CYP1B1 gene rs1056836 polymorphism and risk of PCa was not significant (AB vs. AA, OR = 1.03, 95% CI = 0.96–1.12, *P*=0.401).

#### Analysis of the CYP1B1 rs10012, rs162549, rs1056827, rs1056837, rs1800440, rs2551188, and rs2567206 polymorphisms

The meta-analysis for associations between the rs10012, rs162549, rs1056827, rs1056837, rs1800440, rs2551188, and rs2567206 polymorphisms and PCa risk in the overall population included three, two, five, two, four, four, and four independent studies, respectively. Due to significant between-study heterogeneity indicated by the *Q*-test, rs1056827, rs1056837, and rs2567206 were analyzed using the random-effect model. The fixed model was used to analyze the rs10012, rs162549, rs1800440, and rs2551188 polymorphisms. Overall, no significant association was detected between these SNPs and PCa risk (rs10012, G vs. C, OR = 1.05, 95% CI = 0.91–1.21, *P*=0.508; rs162549, T vs. A, OR = 1.07, 95% CI = 0.96–1.19, *P*=0.226; rs1800440, G vs. A, OR = 0.95, 95% CI = 0.85–1.06, *P*=0.374; rs2551188, T vs. C, OR = 0.96, 95% CI = 0.87–1.07, *P*=0.474).

### Evaluation of publication bias

**Figure 3:**
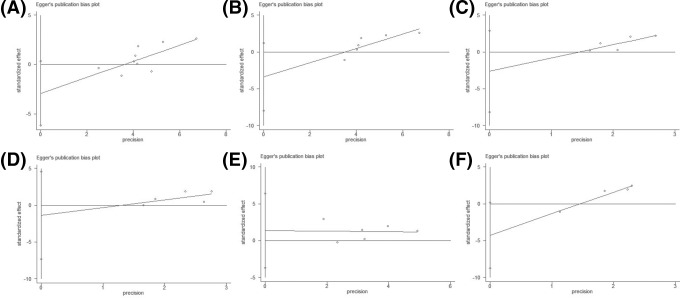
Egger’s publication bias analysis Egger’s publication bias plot for rs1048943 polymorphism in overall population (B vs. A, (**A**)) and Asian population (B vs. A, (**B**); BB vs. AA, (**C**); BB vs. AA+AB, (**D**)), for rs4646903 polymorphism in Asian population (AB vs. AA, (**E**)), and Caucasian population (BB vs. AA, (**F**)). Abbreviation: vs., versus.

**Figure 4:**
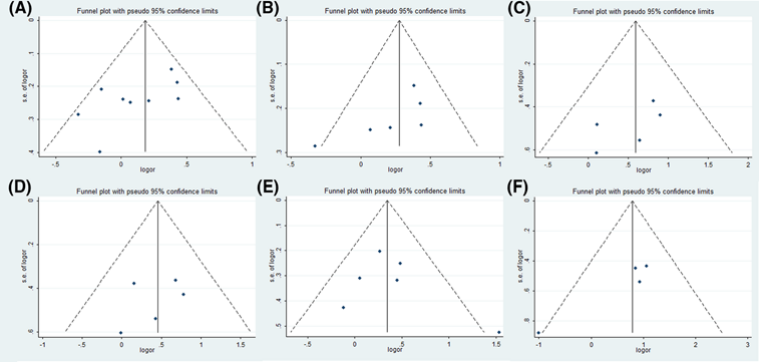
Funnel plots for statistically significant meta-analysis Funnel plots for rs1048943 polymorphism in overall population (B vs. A, (**A**)) and Asian population (B vs. A, (**B**); BB vs. AA, (**C**); BB vs. AA+AB, (**D**)), for rs4646903 polymorphism in Asian population (AB vs. AA, (**E**)), and Caucasian population (BB vs. AA, (**F**)). Abbreviation: vs., versus.

Egger’s linear regression test was used to evaluate any publication bias by funnel plot asymmetry (Table 3). The intercept provides an assessment of asymmetry—more deviation from zero indicates more asymmetry. Egger’s linear regression test provided evidence of publication bias for the rs1048943 polymorphism in the overall population (AB vs. AA: *t* = −2.95, *P*=0.021; BB + AB vs. AA: *t* = −3.53, *P*=0.010) and in the Asian populations (BB + AB vs. AA: *t* = −3.15, *P*=0.035). Egger’s publication bias plots and Funnel plots for positive results are shown in Figures 3 and 4, respectively. No publication bias was found in other analyses.

**Table 3:** Egger’s linear regression test to measure the funnel plot asymmetric

Polymorphisms	Y axle intercept: a (95% CI)				
	B vs. A	BB vs. AA	AB vs. AA	BB + AB vs. AA	BB vs. AA + AB
rs1048943 (Overall)	−2.93 (−6.19, 0.33)	0.41 (−3.32, 4.14)	−2.75 (−4.95, −0.54)*	−2.89 (−4.82, −0.96)*	0.533 (−2.45, 3.51)
rs1048943 (Asian)	−3.39 (−7.96, 1.18)	−2.63 (−8.19, 2.92)	−2.52 (−5.26, 0.22)	−2.75 (−5.18, −0.33)*	−1.38 (−7.33, 4.56)
rs4646903 (Asian)	2.35 (−2.77, 7.47)	0.74 (−1.30, 2.79)	1.378 (−3.65, 6.40)	1.69 (−4.03, 7.42)	0.59 (−1.04, 2.21)
rs4646903 (Caucasian)	−7.21 (−17.40, 2.98)	−4.27 (−8.73, 0.19)	−1.64 (−31.65, 28.37)	−3.49 (−30.55, 23.57)	−3.46 (−6.38, −0.54)*

* *P*<0.05

## Discussion

In recent years, genetic susceptibility to cancer has been a hotspot of research in the scientific community. Emerging evidence has demonstrated the potential association between gene polymorphisms and cancer risk, particularly in the CYP1 family including CYP1A1 and CYP1B1. For instance, CYP1A1 polymorphisms are known to be associated with susceptibility to a wide variety of cancers including lung [44], bladder [45], pancreatic [46], and breast cancers [47]. Similarly, the association of CYP1B1 polymorphisms and the risk of several types of cancer has been explored [48–50]. Currently, the link between CYP1 family gene polymorphisms and PCa susceptibility is attracting widespread attention. Numerous studies have focussed on the relationship between CYP proteins and their SNPs, as well as their possible effects on the development of PCa. Both CYP1A1 and CYP1B1 are involved in the metabolism of numerous carcinogens and steroidal hormones including estrogens [51,52]. Furthermore, Cavalieri et al. [53] reported that metabolites of chemical carcinogens generated by CYP1B1 catalysis can induce PCa in animal models. The CYP1A1*2A (rs4646903) polymorphism involves a thymidine to cytosine substitution at position 3801 of the 3′-non-coding region downstream of the polyadenylation site [54]. Although some studies have shown that the rs4646903 variant significantly increases the activity of mutant enzymes, the results are conflicting [55]. The CYP1A1*2B (rs1048943) polymorphism is the second most common CYP1A1 polymorphism, which involves an adenine to guanine transition at position 2455 of codon 462 at exon 7 [56]. It has been reported that the rs1048943 polymorphism induces an increase in CYP1A1 at the mRNA level [57], and Kisselev et al. [58] reported that this variant significantly increases the catalytic activity for all hydroxylation sites toward substrates including 17β-estradiol (E2) and estrone (E1), most significantly for 2-hydroxylation. The CYP1B1*3 (rs1056836) polymorphism is located in the third exon, and can result in a leucine to valine substitution [59]. This variant has been associated with higher hydroxylation activity, which may consequently increase the risk of various forms of cancer including PCa [51]. These results suggest that polymorphisms of CYP1A1 and CYP1B1 might play a role in promoting tumorigenesis by changing the activity of hydroxylase in the process of estrogen hydroxylation to 2-hydroxyestrogen (2-OH HE) and 4-OH HEs. However, the conclusions of meta-analyses of the association between CYP1A1 and CYP1B1 polymorphisms and PCa risk have been inconsistent [6,9,10,60], and so we endeavored to conduct a systematic meta-analysis to precisely estimate this association, and provide a more comprehensive and reliable conclusion.

The present study provides a systematic analysis of all available case–control studies on the CYP1A1 rs1048943 and rs4646903; and CYP1B1 rs1056836, rs10012, rs162549, rs1056827, rs1056837, rs1800440, rs2551188, and rs2567206 polymorphisms and the risk of PCa. Previously, no significant associations have been found between the CYP1B1 rs10012, rs162549, rs1800440, and rs2551188 polymorphisms and PCa risk in the overall population. A possible explanation is that PCa is a multigenic disease, and the effect of a single polymorphism may therefore be limited. On the other hand, our study demonstrated that the rs1048943 polymorphism was associated with PCa susceptibility in the overall population. Results were similar in the Asian populations; however, no effect was detectable in men of Caucasian descent. Furthermore, our meta-analysis provided evidence that the rs4646903 polymorphism in the AB genotype compared with AA, and BB genotype compared with AA were considered as risk factors for PCa in Asian and Caucasian populations, respectively. However, the CYP1A1 rs4646903 polymorphism was not associated with an increased risk of PCa overall. The effect of polymorphisms on PCa susceptibility is influenced by ethnicity. Data from the National Central for Biotechnology Information (NCBI) show that the B allele (the minor allele) of rs4646903 is present in 34.0% of the Asian population, 10.7% of the Caucasian population, and 23.4% of the African-American population. However, African Americans are reported to have the highest incidence of PCa, which not only emphasizes the racial background of the disease [61], but also confirms the interaction between genetic and environmental factors in PCa. The interaction of genetic and lifestyle factors including dietary fat, obesity, and sexual factors could explain these ethnic differences to some extent. Undoubtedly, a larger study would provide more insight into the association of the rs1048943 and rs4646903 polymorphisms with PCa susceptibility in different ethnicities, especially African Americans.

A meta-analysis performed by Zhang et al. [60] showed that the CYP1B1 rs1056836, rs1800440, and rs1056827 polymorphisms were associated with susceptibility to PCa, which contradicts the results of Cui et al. [9] who found no association between the CYP1B1 rs1056836 polymorphism and PCa risk in the overall and Caucasian populations. No significant association with PCa susceptibility was detected for rs1800440 in our study; however, we understand that with much larger populations and more updated studies, our results may be more reliable. In agreement with our results, Ding et al. [6] found that the CYP1A1 rs4646903 polymorphism was responsible for increasing the risk of developing PCa. Li et al. [10], on the other hand, reported that the rs1048943—and not rs4646903—polymorphism was associated with PCa susceptibility, in line with the results of the present analysis.

Between-study heterogeneity is one of the pivotal issues affecting the results of the present meta-analysis. Subgroup analysis based on ethnic groups revealed a decrease in the heterogeneity amongst studies. These results indicate that ethnic subgroups contribute significantly to the high heterogeneity observed in our analysis. Due to the limited studies of hospital-based controls and genotyping methods other than PCR-RFLP, we were unable to conduct subgroup analysis based on the source of the controls and genotyping methods. However, their impact on heterogeneity should not be ignored. It is well known that using different detection methods to determine genotypes increases the heterogeneity between studies. Therefore, additional studies with different sources of control groups and genetic methods are still necessary in order to assess the impact of these polymorphisms on PCa risk. Studies that include hospital-based control groups and genotyping methods other than PCR-RFLP such as AS-PCR, Taqman, and Genechips are particularly important. Publication bias was observed in some comparisons of the present meta-analysis, which may be because the present study only included English and Chinese publications, possibly resulting in selective bias.

There are several potential limitations of this meta-analysis that should be considered. First, patients with benign prostatic hyperplasia (BPH) were enrolled as controls in our study, which may have an effect on the final results. Second, accurate determination of the association between the CYP1B1 rs1056836 polymorphism and the risk of PCa was not possible due to significant heterogeneity in the overall, Caucasian, and Asian populations. Third, our results were based on unadjusted estimates. A more reliable estimate could have been achieved if individual data such as age and other environmental factors were available.

## Conclusion

To summarize, this meta-analysis suggests that the CYP1A1 rs1048943 and rs4646903 polymorphisms are associated with PCa risk, especially in the Asian and Caucasian populations, respectively. No significant associations were detected for the CYP1B1 rs10012, rs162549, rs1800440, or rs2551188 polymorphisms. Larger studies are necessary to verify our findings and elucidate the exact association between CYP1A1 and CYP1B1 polymorphisms and susceptibility to PCa.

## Abbreviations

AS-PCR: allele-specific PCR
CI: confidence interval
CNKI: China National Knowledge Infrastructure
CYP: cytochrome P450
CYP1A1: cytochrome P450 1A1
CYP1B1: cytochrome P450 1B1
NOS: Newcastle–Ottawa Scale
OR: odds ratio
PCa: prostate cancer
PCR-RFLP: PCR–restriction fragment length polymorphism
SNP: single nucleotide polymorphism
